# Proton metabolic mapping of the brain at 7 T using a two‐dimensional free induction decay–echo‐planar spectroscopic imaging readout with lipid suppression

**DOI:** 10.1002/nbm.4771

**Published:** 2022-05-26

**Authors:** Kyung Min Nam, Arjan D. Hendriks, Vincent O. Boer, Dennis W. J. Klomp, Jannie P. Wijnen, Alex A. Bhogal

**Affiliations:** ^1^ Center for Image Sciences, Department of Radiology University Medical Centre Utrecht Utrecht; ^2^ Danish Research Centre for Magnetic Resonance, Centre for Functional and Diagnostic Imaging and Research Copenhagen University Hospital Hvidovre Hvidovre Denmark

**Keywords:** 7 T, crusher coil, EPSI, FID, L2‐regularization, lipid suppression, MRSI, pulse acquire

## Abstract

The increased signal‐to‐noise ratio (SNR) and chemical shift dispersion at high magnetic fields (≥7 T) have enabled neuro‐metabolic imaging at high spatial resolutions. To avoid very long acquisition times with conventional magnetic resonance spectroscopic imaging (MRSI) phase‐encoding schemes, solutions such as pulse‐acquire or free induction decay (FID) sequences with short repetition time and inner volume selection methods with acceleration (echo‐planar spectroscopic imaging [EPSI]), have been proposed. With the inner volume selection methods, limited spatial coverage of the brain and long echo times may still impede clinical implementation. FID‐MRSI sequences benefit from a short echo time and have a high SNR per time unit; however, contamination from strong extra‐cranial lipid signals remains a problem that can hinder correct metabolite quantification. L2‐regularization can be applied to remove lipid signals in cases with high spatial resolution and accurate prior knowledge. In this work, we developed an accelerated two‐dimensional (2D) FID‐MRSI sequence using an echo‐planar readout and investigated the performance of lipid suppression by L2‐regularization, an external crusher coil, and the combination of these two methods to compare the resulting spectral quality in three subjects. The reduction factor of lipid suppression using the crusher coil alone varies from 2 to 7 in the lipid region of the brain boundary. For the combination of the two methods, the average lipid area inside the brain was reduced by 2% to 38% compared with that of unsuppressed lipids, depending on the subject's region of interest. 2D FID‐EPSI with external lipid crushing and L2‐regularization provides high in‐plane coverage and is suitable for investigating brain metabolite distributions at high fields.

## Graphical abstract

We investigated the performance of lipid suppression by L2‐regularization, an external crusher coil, and the combination of these two. FID‐EPSI with external lipid crushing and L2‐regularization provides high in‐plane coverage and is suitable for investigating brain metabolite distributions at high fields.

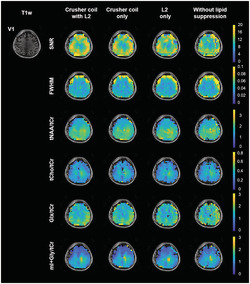

**Abbreviations**
^1^H MRSI: proton MR spectroscopic imaging
Cho: choline
Cr: creatine
CSDE: chemical shift displacement error
ECC: eddy current correction
EPI: echo‐planar imaging
EPSI: echo‐planar spectroscopic imaging
FID: free induction decay
FOV: field of view
FWHM: full width at half maximum
Gln: glutamine
Glu: glutamate
Glx: glutamate‐glutamine
Gly: glycine
GM: gray matter
mI: myo‐inositol
NAA: N‐acetyl aspartate
NAAG: N‐acetyl aspartate glutamate
OVS: outer volume suppression
PA: pulse acquire
PC: phase correction
PE MRSI: phase‐encoded MRSI
PRESS: position‐resolved spectroscopy
SAR: specific absorption rate
semi‐LASER: semilocalization by adiabatic selective refocusing
SNR: signal‐to‐noise ratio
STEAM: stimulated echo acquisition mode
T1w: T1‐weighted
TE: echo time
TR: repetition time
VAPOR: variable power and optimized relaxation delays
WM: white matter

## 1 INTRODUCTION

Magnetic resonance spectroscopic imaging (MRSI) is a noninvasive technique for measuring and visualizing neuro‐chemical distributions. Ultra‐high field MR scanners (≥7 T) are appealing tools for MRSI acquisition because signal‐to‐noise ratio (SNR) and chemical shift dispersion increase as a function of magnetic field strength.^1^ These properties enable MRSI with increased spatial resolution and more resolved metabolite resonances for a given scan time compared with MRSI acquisition at lower field. Nevertheless, MRSI at high field is challenging, and straightforward and robust solutions are still missing to reduce the acquisition time and avoid lipid signal contamination.

The relatively low spatial sampling associated with conventional MRSI leads to a broad point‐spread function (PSF) that can induce far‐reaching signal contamination. This phenomenon, referred to as signal leakage or voxel bleeding, poses problems, in particular considering the strong lipid signals in the skull that can permeate central brain regions. A broad lipid PSF can lead to unwanted artifacts that impede spectral quality and hinder accurate metabolite quantification. Moving to higher spatial resolutions (e.g., ≥ 64 x 64)^2^, ^3^ can mitigate this effect. However, this comes at the cost of a prohibitively long scan time. For this reason, a strong interest in developing fast MRSI acquisition techniques has emerged; however, the need for effective solutions to the lipid contamination problem remains.

Several volume localization and extra‐cranial lipid signal suppression techniques have been developed to address the lipid contamination problem. These include inner volume selection (IVS) techniques that prevent excitation of extra‐cranial/skull tissue and are insensitive to lipid artifacts when planned correctly. The IVS technique typically utilizes RF pulses to localize the spin‐echo signal to the volume of interest. Diverse sequences based on this localization method using RF pulses have been proposed, such as stimulated echo acquisition mode (STEAM^4^), point‐resolved spectroscopy (PRESS^5^, ^6^), or localization by adiabatic spin‐echo refocusing (LASER/sLASER^7^, ^8^, ^9^). The main drawbacks of IVS techniques, and which are also factors that impede clinical application, are limited spatial coverage, relatively long echo time (TE), and long repetition time (TR). The latter is driven by the specific absorption rate (SAR) constraint that determines average allowable RF power deposition. Nonspatially selective methods provide an alternative approach whereby signals from lipid‐rich regions are crushed after excitation and before signal readout. To achieve this lipid suppression, additional hardware elements are required. Examples include additional insert coils to perform local signal dephasing, that is, “crusher coil”,^10^ or external shim coils capable of producing steerable second‐order spherical harmonic fields for controlled signal dephasing.^11^ These methods can be combined with optimized RF pulse sequences for enhanced outer volume suppression (OVS). Recently, elliptical localization with pulsed second‐order fields (ECLIPSE^11^, ^12^) was demonstrated as a hybrid model using IVS, OVS, and dedicated shim coils. In addition, lipid artifacts can also be removed during postprocessing^13^, ^14^, ^15^ using methods such as the L2‐regularization algorithm.^14^, ^15^

Free induction decay (FID) ^1^H MRSI^2^, ^16^ has been introduced as a method that can overcome several limitations associated with IVS (i.e., limited spatial coverage, the relatively long TE and TR). For spatial localization, conventional phase‐encoded MRSI^17^ is considered the gold standard in terms of sensitivity and localization performance. Because each spatial dimension requires a phase‐encoding step, this approach is time‐consuming for high‐resolution acquisitions with one voxel per TR. Considering these criteria, high‐speed FID‐MRSI is desirable to reduce scan time, expand volume coverage, and decrease motion sensitivity while considering SNR. One of the common readout schemes for accelerating MRSI acquisition involves the use of rapidly alternating echo‐planar gradients (echo‐planar spectroscopic imaging [EPSI]) to encode several k‐space locations in a single TR. Because of inconsistencies between odd and even lines in k‐t space, the EPSI readout leads to a spectral ghosting artifact. This artifact can be reduced by separately processing the odd and even echoes at the cost of a 50% reduction in spectral bandwidth. Improvements on the EPSI scheme have been proposed including flyback EPSI,^18^ temporal interleaving,^19^ the interlaced Fourier transform,^20^ the Fourier shift theorem,^21^ and reversed readout gradients.^22^, ^23^ In this work, we applied a phase correction method, commonly used for the ghosting artifact correction^24^ in echo‐planar imaging (EPI) reconstruction, to the EPSI data. We used the phase information from a fully encoded water reference scan.

Considering the current limitations for MRSI at high field, this study aimed to enable a fast acquisition method for brain metabolite mapping with increased spatial resolution and extensive in‐plane coverage, and direct lipid suppression at 7 T. We combined FID with an EPSI readout and non‐RF–based lipid signal suppression using a crusher coil.^10^ Furthermore, we investigated the additional effect of using the L2‐regularized algorithm^14^ to further residual lipid signal suppression during postprocessing. With this approach, we minimized SAR and maximized the amount of spectral data available, particularly including at the boundary regions of the brain.

## 2 METHODS

### 2.1 Experimental setup and protocol

This study was approved by the local ethical committee of the University Medical Center Utrecht. Data were acquired from three healthy volunteers (one male aged 39 years, and two females aged 40 years) upon providing informed consent. Experiments were conducted using a 7 T MR scanner (Philips Medical Systems, Best, The Netherlands) equipped with a two‐channel RF transmit coil and a 32‐channel head‐receive coil (Nova Medical, Wilmington, MA). The gradient system provided a maximum strength of 40 mT/m and a maximum slew rate of 200 mT/m/ms. Image‐based shimming^25^ was performed using third‐order spherical harmonic terms with the exception of the Z^3^ term. The Z^3^ amplifier has a dynamic range of ±10A and was used to pulse the crusher coil for 1.7 ms between excitation and readout. To ensure subject safety, a 1.25A fuse was fitted in the cable between the amplifier and the crusher coil to prevent overheating of the coil windings. For more details, see Boer et al.^10^ Anatomical imaging consisted of a three‐dimensional (3D) T_1_‐weighted inversion recovery‐prepared spoiled gradient echo acquisition with the following parameters: field of view (FOV) = 224 x 224 x 128 mm^3^, voxel size = 2 x 2 x 2 mm^3^, TE/TR = 1.8/4 ms, TI = 1300 ms.

### 2.2 Sequence design and acquisition

**FIGURE 1:**
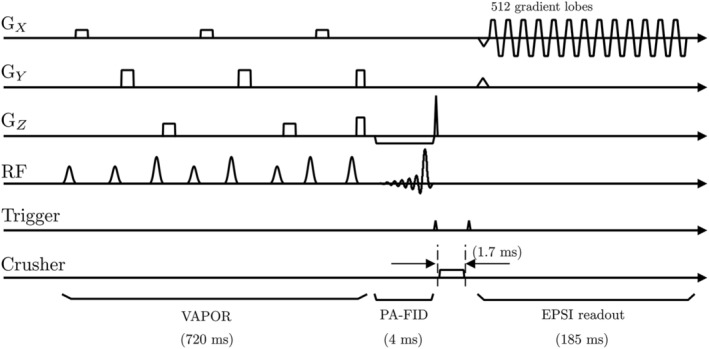
Pulse sequence diagram used for the free induction decay–echo‐planar spectroscopic imaging (FID‐EPSI) acquisition. The sequence consists of variable power and optimized relaxation delays (VAPOR) water suppression and the trigger signal generated by spectrometer initialization of an external amplifier that drives the crusher coil, followed by the FID‐EPSI readout at 7 T. The duration of the lipid suppression gradient is 1.7 ms. The FID‐EPSI sampled 512 spectral points with a 2379 Hz spectral bandwidth and a 185 ms readout time

The sequence diagram for the VAPOR^16^, ^26^, ^27^ water suppression and the two‐dimensional (2D) FID‐EPSI acquisition with lipid suppression is shown in Figure 1, including the time to live (TTL) trigger timing (i.e., 1.7 ms) used to pulse the crusher coil. The VAPOR water suppression (duration = 750 ms) module used eight water‐suppression pulses with variable flip angles. For each acquisition, a nonwater‐suppressed (NWS) reference was acquired using similar acquisition parameters as its water‐suppressed counterpart except with the number of averages adapted to two. The FID‐EPSI data were sampled with two spatial dimensions and one spectral dimension. The echo‐planar readout dimension consisted of 512 alternating trapezoidal gradient shapes. Data were acquired during the gradient plateaus (i.e., 0.2529 ms per gradient lobe). A real‐time frequency drift correction option (vendor‐supplied) provided frequency stabilization^23^, ^28^ during the EPSI acquisition. To examine the effect of lipid signal contamination in the central and cortical regions of the brain, two different datasets were acquired: (1) FID‐EPSI readout including direct lipid suppression using the crusher coil; and (2) FID‐EPSI without direct lipid suppression. For FID‐EPSI acquisitions, spectral bandwidth, TR, flip angle, and acquisition matrix were chosen such that spatial coverage and the effects of lipid signal suppression could be optimally compared. Slice selection was identical for all acquisitions. The acquisition parameters were: matrix size = 38 x 38, FOV = 228 x 228 mm^2^, slice thickness = 12 mm, TE/TR = 3/960 ms, spectral bandwidth = 2379 Hz, 16 signal averages for the water‐suppressed scan with a total scan duration of 9 min 45 s and two signal averages for the NWS reference scan with a total scan duration of 1 min 14 s.

### 2.3 Phantom measurements for validation of EPSI ghosting artifact correction

**FIGURE 2:**
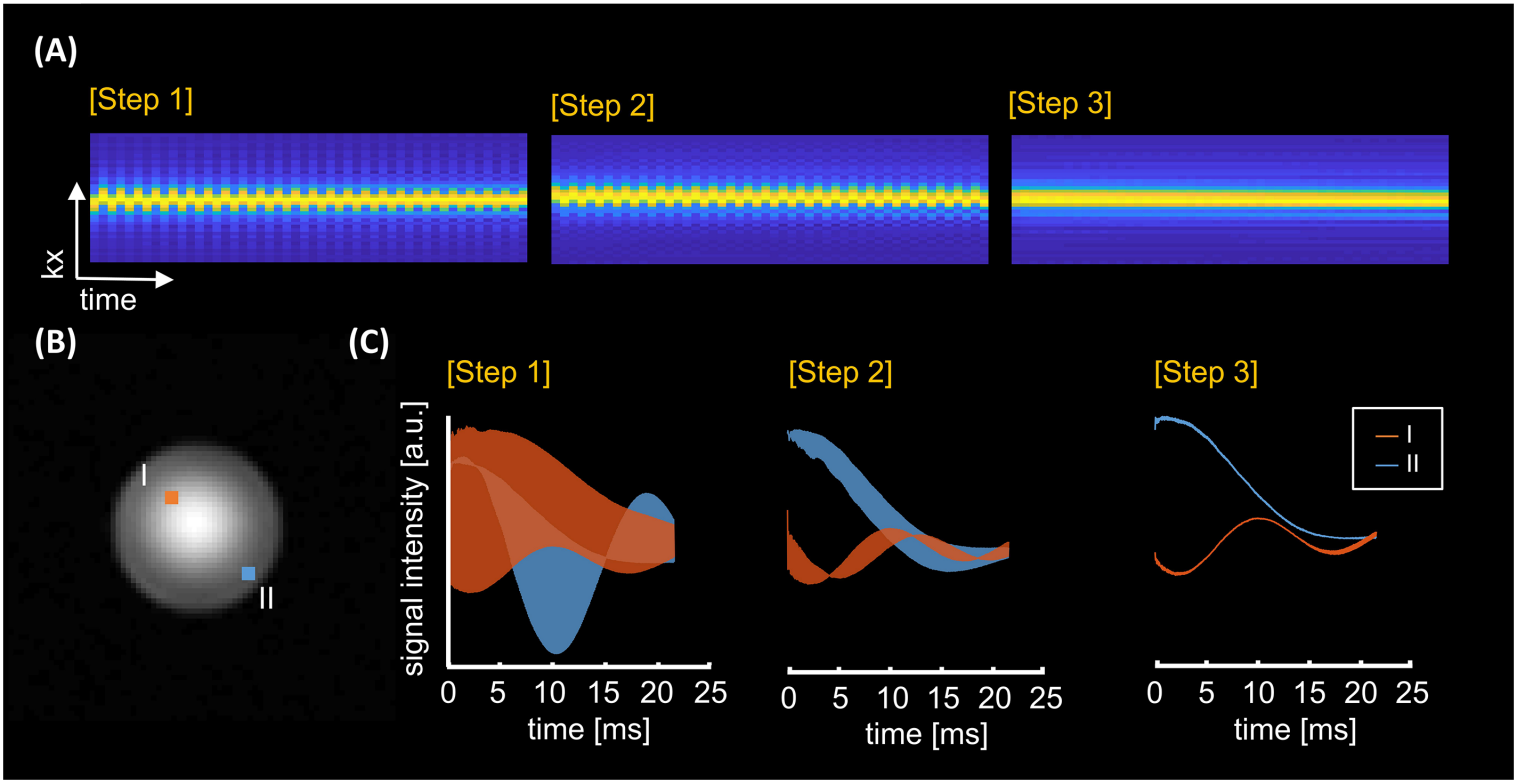
Spectral ghosting procedure for the echo‐planar spectroscopic imaging (EPSI) data via reconstruction steps (see Table 1). Gradient‐echo image (B) showed using a calibration phantom A (a sphere of 10 cm diameter) placed in the isocenter of the MR bore. (A) The effect on the acquired k‐t signal of the free induction decay (FID)‐EPSI readout for the phase‐corrected data by the following steps at the center location in (B). The alignment of k‐t space (A) is shown using the water reference signal, and (C) FID signals are presented at locations I and II in (B)

A calibration phantom (defined as phantom A) containing 5 ml of acetate, 10 ml of ethanol, 8 ml of phosphoric acid, and 1 ml of aqueous solution with 120 mg/ml CuSO_4_, was used to demonstrate fluctuations^29^ of the k‐t signal during the EPSI readout before and after correction for the Nyquist ghosting artifact. For validation, phantom experiments were performed to visualize the effect of the echo alignment on the k‐t signal of the EPSI water reference (Figure 2).

### 2.4 EPSI data reconstruction

All data reconstruction and postprocessing steps were performed using inhouse‐developed MATLAB scripts (MathWorks, Natick, MA). A series of reconstruction steps are shown in Table 1. The signal averaging of EPSI data was performed in the first step. The second step consisted of flipping k‐space lines corresponding to odd echoes along the $k_{x}$ direction. The Nyquist ghosting artifact,^24^ which is caused by misalignment between odd and even echo lines of $k_{x}$, was addressed by correcting the phase at each readout line in the third step. The phase difference term $\varphi_{\text{diff}}$ was calculated from the water reference signal, which was separately measured. The phase correction term $\varphi_{\text{corr}}$ for echo alignment of k‐t space was approximated by averaging the phase difference $\varphi_{\text{diff}}$, which shifts the positions of the odd and even echoes from the reference signals. The phase‐corrected EPSI data were calculated by multiplying the linear phase correction term $\varphi_{\text{corr}}$. To reduce the side lobes of the PSF, a 2D Hamming window filter was used in the fourth step. The final step included the Roemer equal noise algorithm for channel combination.^30^, ^31^, ^32^ Here, the noise matrix^33^ Ψ was applied to decorrelate the noise‐correlated signals between the receiver channels^34^ for optimal SNR. The noise samples $n_{s}$ were obtained from the raw data of an anatomical MR image with low resolution (i.e., 6 × 6 mm^2^). The sensitivity information S was obtained from the water reference signal.

**TABLE 1:** The reconstruction algorithm in the free induction decay–echo‐planar spectroscopic imaging data

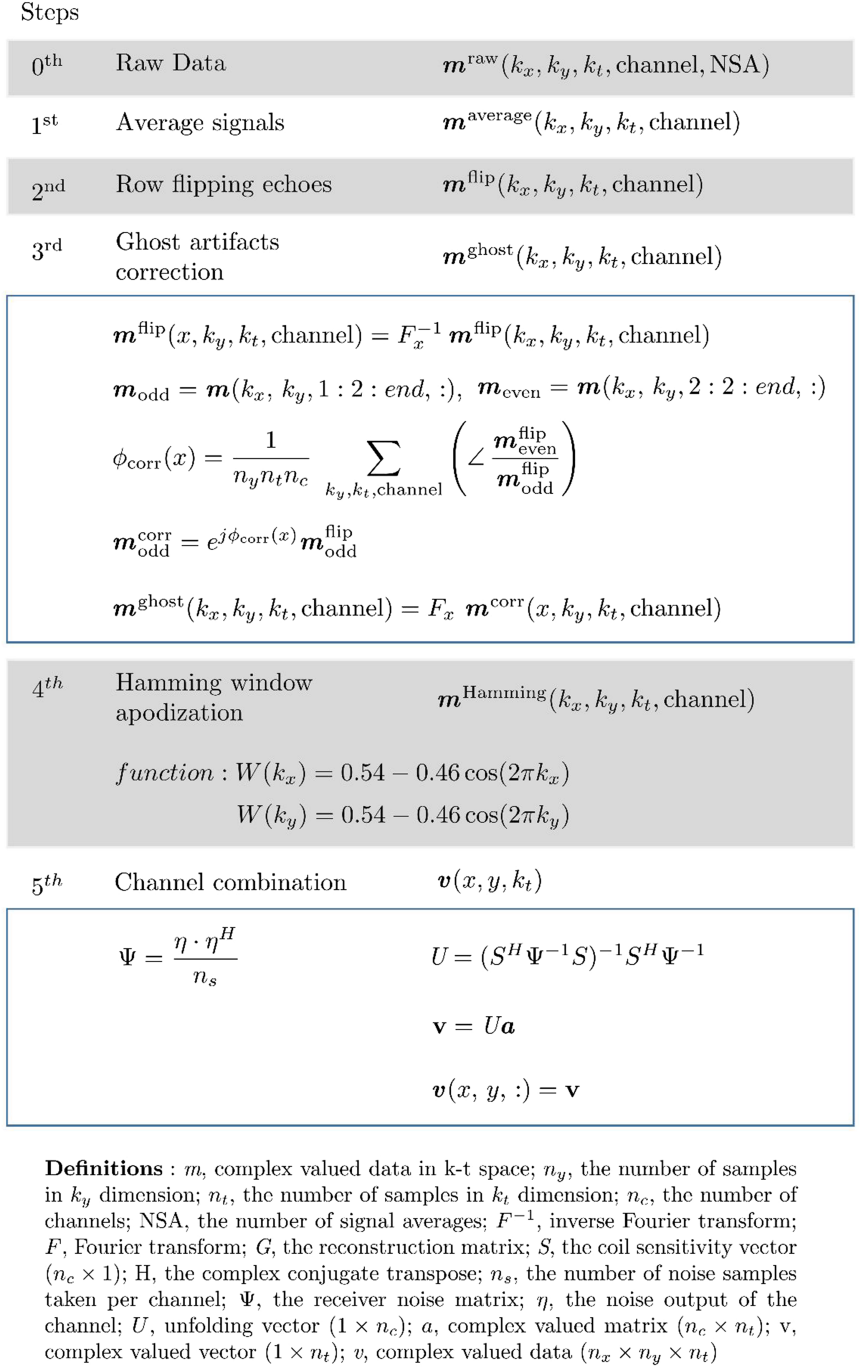

### 2.5 Postprocessing

**FIGURE 3:**
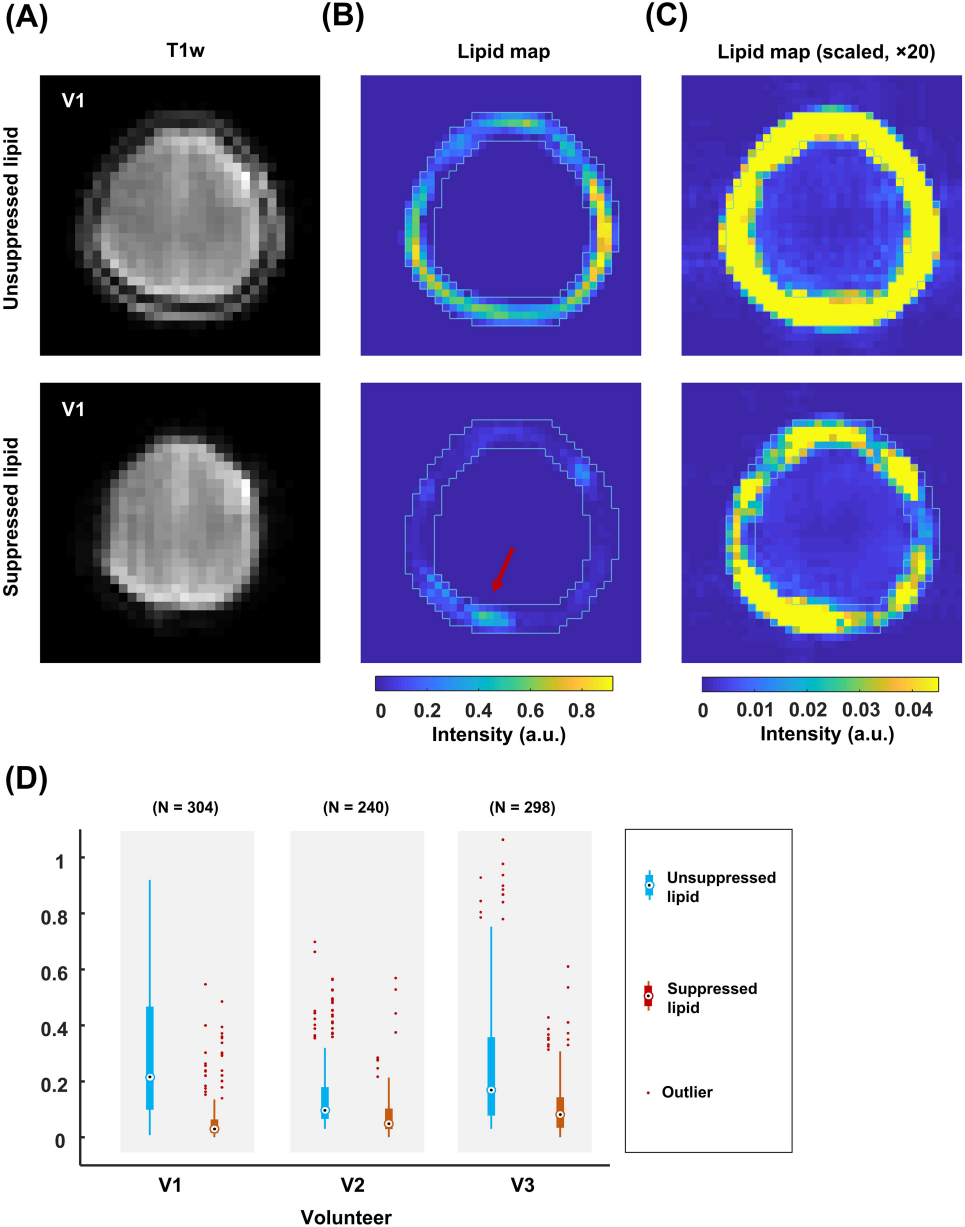
(A) Low resolution of T_1_‐weighted images with/without lipid suppression using a crusher coil. (B, C) Lipid contamination maps in a matrix of 38 × 38 voxels. The lipid maps are calculated by integrating the spectrum between 0.8 and 1.6 ppm. The lipid binary mask (light blue line) in the lipid contamination map without lipid suppression (B, top) is used to the map (B, bottom) with lipid suppression. The area of the residual lipids (red arrow) is indicated in the lipid mask. (C) Maps with a factor of 20 multiplied intensity scale visualize lipid signal leakage near the skull in more detail. A point‐spread function pattern is visible in the unsuppressed case (C, top). This pattern is reduced in the suppressed case, where the lipid suppression reduction factor was 7 in V1, and 2 in V2 and V3. (D) Distribution of the (un)suppressed lipid signal intensity of the voxels in the entire region of interest per volunteer

Further processing steps included eddy current correction,^35^ first‐order phase correction using a linear prediction model, spectral water peak alignment, and residual water removal, which was executed via the fast Hankel–Lanczos variant.^1^, ^36^, ^37^ After removing the residual water signals, a lipid contamination map was generated by integrating spectral signals ranging between 0.8 and 1.6 ppm in the lipid (un)suppressed acquisitions of the FID‐EPSI sequence. Taking the unsuppressed lipid map as a reference, the lipid suppression factor was calculated to evaluate the performance of the combination of FID‐EPSI and the crusher coil in Figure 3. For the implementation of the L2‐regularization algorithm,^15^, ^38^ we used the open‐source toolbox for MATLAB, which is available via the website (https://martinos.org/~berkin/software.html). We compared the performance of lipid suppression in the case of (a) FID‐EPSI with direct lipid suppression (i.e., crusher coil); (b) FID‐EPSI with L2‐regularization; and (c) FID‐EPSI with direct lipid suppression and L2‐regularization. The lipid binary mask was generated from the lipid contamination map with an arbitrary threshold value using the lipid‐unsuppressed acquisition. The two datasets required in the L2‐regularization algorithm were derived from the water‐suppressed acquisition. The 16 averages were used to obtain the low spatial frequency content, and the two averages were used to generate a lipid image for the dual‐density reconstruction^39^, ^40^ merged in the reconstruction pipeline for the L2‐regularization.

### 2.6 Metabolite fitting

Spectra were fit using LCModel software,^41^ and the range of spectral fitting was set between 1.0 and 4.2 ppm. The basis set was simulated using the Bruker Topspin software and included 17 metabolites: creatine+phosphocreatine (tCr), glutamate+glutamine (Glx), N‐acetylaspartate+N‐acetylaspartylglutamate (tNAA), acetate (Ace), alanine (Ala), aspartate (Asp), glycerophosphocholine+phosphocholine + choline (tCho), γ‐aminobutyric acid (GABA), myo‐inositol+glycine (mI + Gly), glutathione (GSH), and taurine (Tau). Note that the basis set was simulated using an ideal 90‐degree RF pulse and excluded gradients, crusher gradients, and relaxation parameters. Metabolite ratios were analyzed (to total creatine), and were not corrected for the differences of T1 and T2 relaxation effects between the different metabolites and the B1 inhomogeneity. Moreover, LCModel‐simulated signals were included: lipid at 1.3 and 2.0 ppm (Lipid13, Lipid20), and macromolecules at 1.2, 1.4, and 1.7 ppm (MM12, MM14, MM17). Metabolite ratio maps were generated for tNAA, tCho, Glx, and mI + Gly. Fitted metabolite values were normalized to tCr. Metabolite values with Cramér–Rao Lower Bounds (CRLB) higher than 25% were discarded. An overview of the results of QA filtration is provided in Table **S1**. For implications and considerations on the use of CRLB in data QA the reader is directed to the work of Kreis.^42^

## 3 RESULTS

A qualitative comparison of the effect of the ghosting artifact correction method (Table 1) is shown in a spherical phantom in Figure 2. Figure 2A shows the alignment of (k‐t) spatial data at each stage of reconstruction for voxels located in the center of the phantom. The ghosting artifact is minimized by correcting the mismatch between odd and even lines in the spatiotemporal signal. The FID signals from two locations (red and blue voxel) after each reconstruction step are shown in Figure 2C.

The effect of external lipid suppression with the crusher coil is shown in Figure 3. The characteristic PSF pattern of the lipid signal resulting from the Fourier reconstruction is visible throughout the brain for the unsuppressed FID‐EPSI acquisition (Figure 3C, top). This pattern was not present in the suppressed case (Figure 3C, bottom), but it was present in the area near the region of the residual lipids. The lipid contamination maps with and without lipid suppression for the two other volunteers are shown in Figure (S4). The signal distribution of the voxels in the lipid mask with and without lipid suppression by the crusher coil is shown in Figure 3D. The median for the signal distribution in the lipid mask is 0.21/0.03 (volunteer 1), 0.09/0.04 (volunteer 2), and 0.16/0.08 (volunteer 3).

**FIGURE 4:**
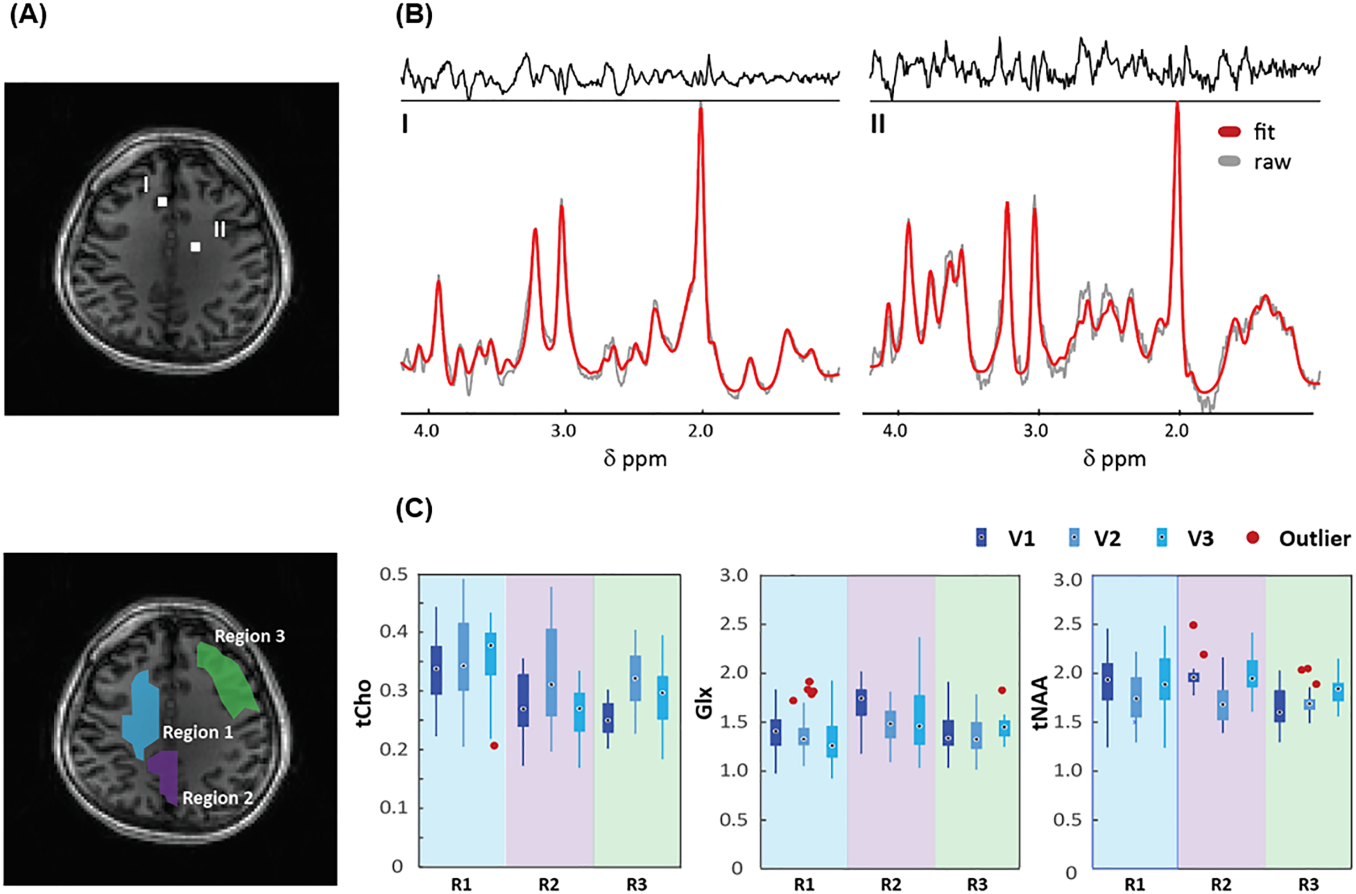
(A) Anatomical brain image with two voxel locations (top) and the three different regions of interest (ROIs) (bottom). (B) MR spectra obtained with crusher coil and L2‐regularization of the two voxels in (A) with LCModel fit. (C) Concentration ratios (tCho, Glx, and tNAA) to total Cr (tCr) as calculated by LCModel for each ROI. The number of voxels of these ROIs (R1/R2/R3) in a matrix size of 38 × 38 is 48/16/38 for volunteer 1 (V1), 52/25/26 for volunteer 2 (V2), and 34/29/27 for volunteer 3 (V3) (see Figure S2)

**FIGURE 5:**
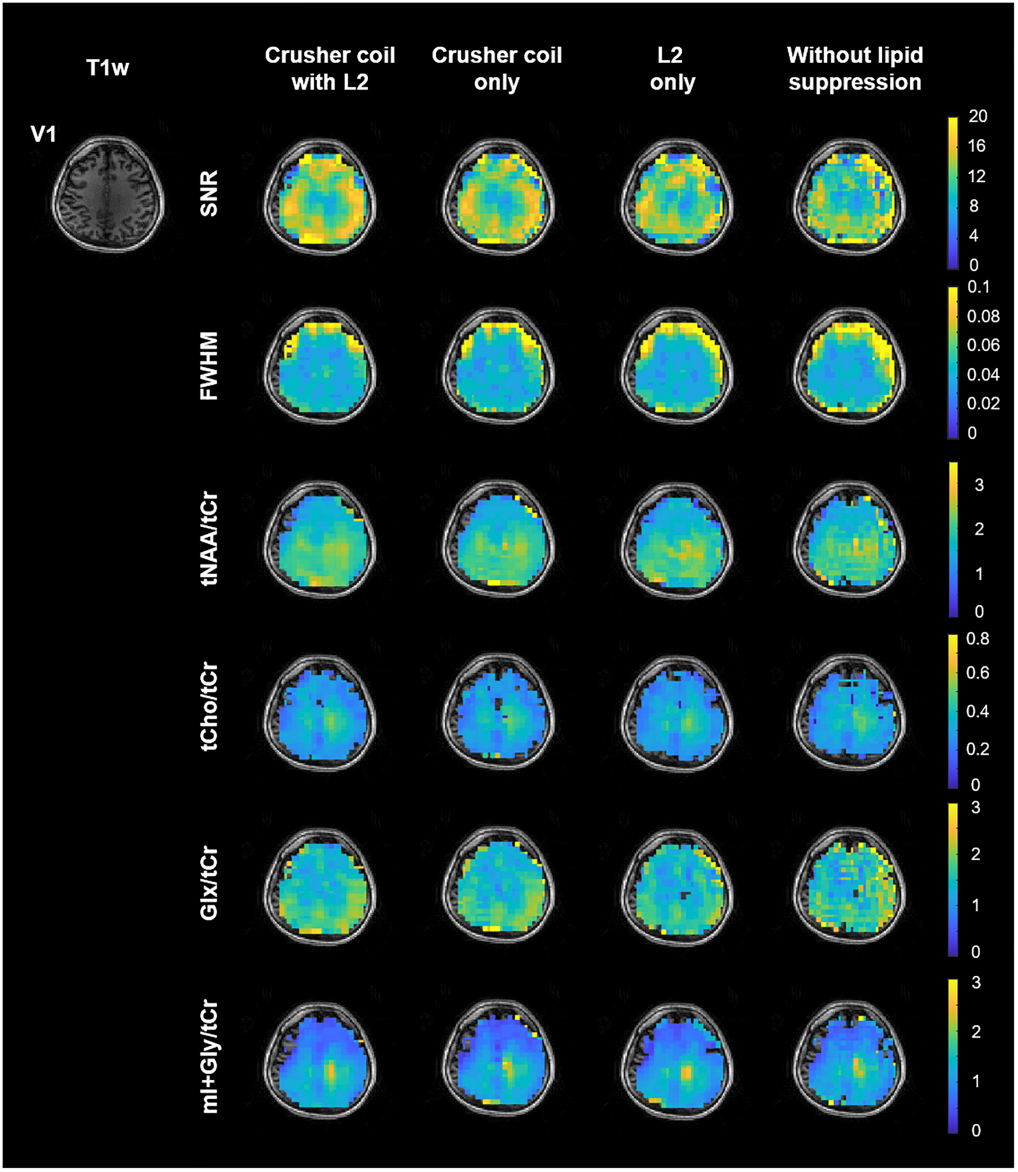
Quality assurance maps (signal‐to‐noise ratio [SNR] and full width at half maximum [FWHM]) and the reconstructed metabolite ratio (divided by creatine + phosphocreatine [tCr]) maps of N‐acetyl‐aspartate + N‐acetyl aspartate glutamate (tNAA), choline + glycerophosphorylcholine + phosphorylcholine (tCho), glutamine + glutamate (Glx), and myo‐inositol + glycine (mI + Gly) were generated using different lipid‐suppression strategies. In the unsuppressed lipid case, clear effects of the lipid point‐spread function were evident for tNAA, Glx, and mI + Gly. Optimal lipid suppression was achieved with the combination of the crusher coil (direct lipid suppression) and L2‐regularization (lipid removal). For visualization, T1‐weighted image and metabolite ratio maps were interpolated by a factor of 2 (the final matrix size is 76 × 76)

Figure 4 shows two individual MR spectra in the brain that demonstrate the effect of lipid signal suppression using the crusher coil in combination with L2‐regularization (see Figure (S5) for the other volunteers). The distribution of metabolite concentration ratios (tCho/tCr, Glx/tCr, and tNAA/tCr) in three different regions of interest (ROIs) of each volunteer are shown in Figure 4C. For the qualitative comparison of FID‐EPSI in combination with the crusher coil and the L2‐regularization algorithm, maps of SNR, full width at half maximum (FWHM), and the metabolite ratio of one volunteer are presented in Figure 5. These maps were calculated based on the LCModel quantification output (metabolite ratio maps for two other volunteers are included in Figure **S1**). The PSF effect of lipids is evident for the four metabolite ratio maps with unsuppressed lipids compared with suppressed lipids. The highest degree of lipid suppression was achieved when combining lipid suppression during acquisition using the crusher coil, followed by L2‐regularization in postprocessing.

**FIGURE 6:**
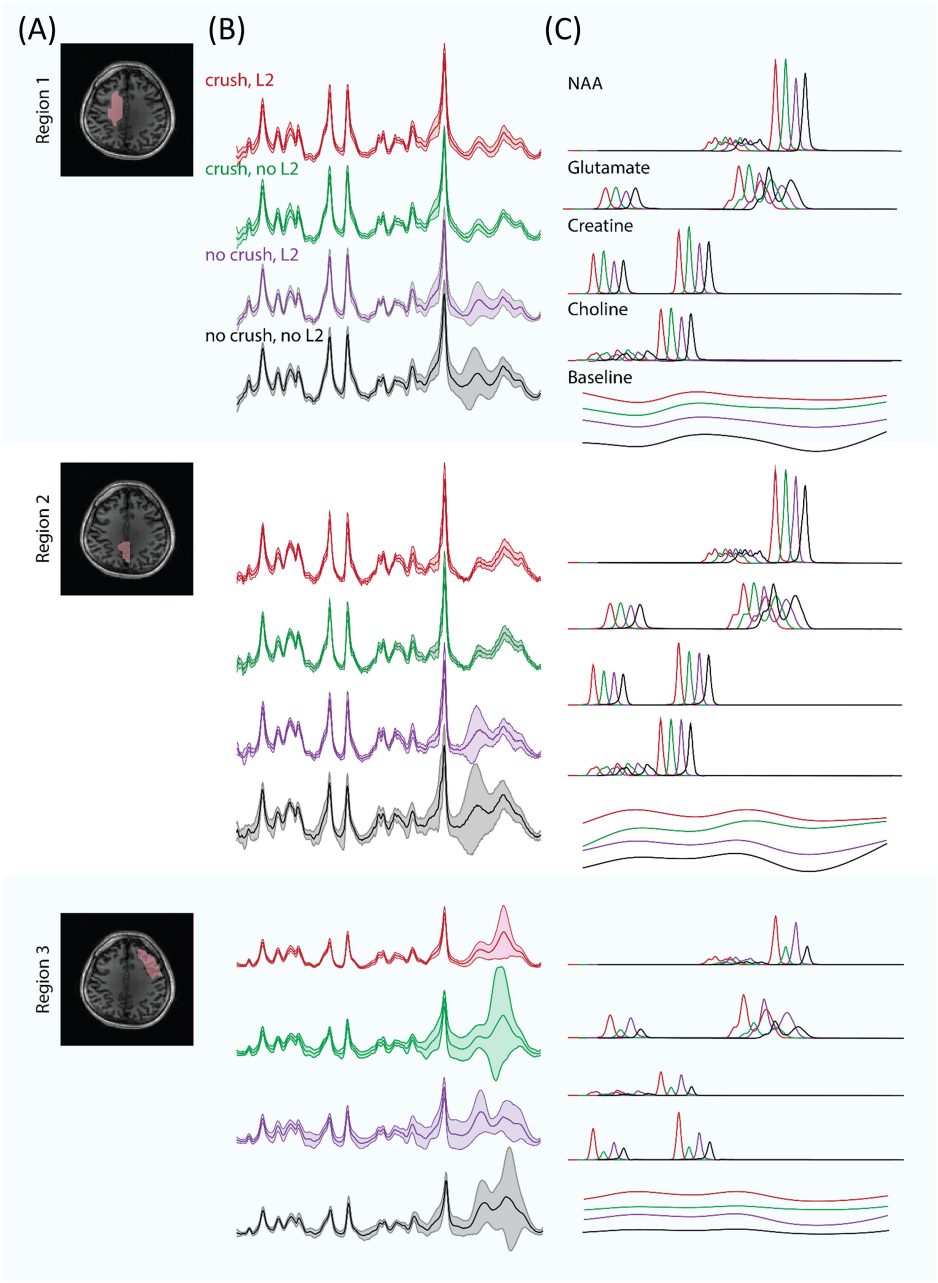
(A) T1‐weighted image with the delineation of three regions of interest (ROIs) at V1. Region 1 contains predominantly white matter, and region 2 contains predominantly gray matter + CSF, while region 3 highlights boundary effects related to the lipid suppression field of the crusher coil. (B) Voxel average (solid line) and standard deviation (shaded area) of the fitted MR spectra for each ROI and each experimental variant; (red) crusher coil and L2‐regularization, (green) crusher coil and no L2‐regularization, (purple) no crusher coil and L2‐regularization, and (black) no crusher coil and no L2‐regularization. (C) Individual fits of tNAA, Glx, tCr, tCho, and the baseline in the four experimental settings (red, green, purple, and black). Note that spectra have been shifted to facilitate visual comparison

For regions 1 and 2 in Figure 6, using the crusher coil seems more effective at lipid suppression than L2‐regularization alone. When calculating the lipid area of ROIs 1 and 2 in V1, the average lipid area of the crusher coil alone was reduced by 12.3% in ROI 1 and 6.8% in ROI 2, compared with the case of L2‐regularization alone. And the average area in the case of combining the two suppression methods decreased by 12.3% in ROI 1 and 9.3% in ROI 2 (Table S2). For all ROIs, the combination of the crusher coil and L2‐regularization resulted in the smallest variation in the MR spectra (shaded areas in Figure 6B). In addition, the fitted signals at regions 1 and 2 were less affected by lipid contaminations than region 3 (i.e., boundary) of the brain. Lipid levels for each ROI per volunteer are reported in Table S2, and the related spectra are visualized in Figure S3.

## 4 DISCUSSION

In this work, we implemented an accelerated ^1^H 2D FID‐MRSI technique that included an EPSI readout with direct lipid suppression during data acquisition using a crusher coil as well as a postprocessing method based on the L2‐regularization approach. This combination directly dephased signals at their extra‐cranial source and removed lipid contamination due to the PSF of signal residues and imperfect crushing. In addition, to address the ghost artifacts associated with EPSI readout, we introduced an EPSI phase correction method, which is a modified version of the correction method described by Schmitt et al.^24^ for EPI. Our findings can be of value to researchers and clinicians interested in accelerated ^1^H MRSI with extensive coverage of the brain, without the use of RF‐based lipid‐suppression techniques. Our work provides a qualitative comparison of MR spectra throughout the brain using different combinations of non‐RF–based lipid suppression. This information can be used to guide MRSI protocols for clinical research studies, particularly at high field.

A strength of our approach with the crusher coil relates to the minimization of SAR‐related TR constraints and the ability to measure metabolites with short T2 (due to our short TE) while having more extensive spatial coverage than IVS methods. However, an associated drawback of the approach to use static magnetic fields for signal crushing is that this technique, in its current implementation, cannot accurately accommodate differences^10^, ^43^, ^44^ in head shape, size, and ROIs (Figure 3C). For example, the reduction factor of lipid suppression varied between 2 and 7 depending on the shape and size of the volunteer's head. This was evinced by the inhomogeneous signal distribution in the lipid map and the variability observed in the lipid suppression values across volunteers (Figure 3D). The distribution of the lipid suppression effect can be evaluated before running the MRSI sequence using a low‐resolution T1‐weighted image acquired while activating the crusher coil. This qualitative assessment can be used to adjust the crusher strength or to change the subject's position in the coil if required and if possible (see Figure (S4)). The fixed geometry of the coil and its applied B_0_ field limit the degrees of freedom to adapt the crushing pattern beyond amplitude scaling. Future iterations of this coil can include more complex winding patterns,^44^ as well as a multichannel design to facilitate tailored lipid suppression. This will allow better field control for improved subject‐dependent suppression, an essential characteristic required for eventual broader application (e.g., clinical application).

Compared with direct suppression, the L2‐regularization method has the advantage of being more convenient to implement because it is performed during postprocessing. Moreover, patient‐specific physiology or geometry do not play a role. Using L2‐regularization alone can provide successful lipid signal suppression (see Figure S3 and Table S2). However, the L2‐regularization method has a significant limitation in that the assumption of orthogonality between the metabolite and lipid signals is not always valid.^15^, ^45^ The consequence of this is that removing lipid signals may also result in the loss of information related to other resonances. Some accelerated 3D FID‐MRSI studies have also reported related baseline distortions.^45^, ^46^, ^47^ This was also evident in one of our example subjects (Figure 6C). In addition, the L2‐regularization method requires the acquisition of additional signal averages to collect the peripheral k‐space information^15^ required for masking the lipid and brain tissue boundaries. This leads to an increase in total scan time. A major issue using the external coil relates to the overcrushing of brain signals in favor of complete suppression. Without direct validation and a more in‐depth study of test–retest reproducibility of the methods presented herein, it remains a somewhat open question as to which lipid suppression (or combination thereof) method is most ideal. To address this shortcoming, an anatomically realistic phantom could be developed that contains representative metabolite distributions surrounded by a lipid shell. In this way, the effect of different suppression strategies could be compared against a ground truth. Nevertheless, for current practical implementation, a complementary L2‐regularization step is attractive in that it can compensate for imperfections in the direct signal crushing or allow a reduction in the strength of the crushing field to better spare tissues of interest.

On the subject of acquisition time, the extension of high fidelity MRSI for whole‐brain applications necessitates acceleration techniques that rely on non‐Cartesian k‐space acquisition. Several flavors have been proposed, including variant EPSI,^48^, ^49^, ^50^, ^51^ spiral,^52^ radial,^53^ concentric ring,^54^, ^55^, ^56^ and rosette^57^, ^58^ k‐space trajectories. For these non‐Cartesian trajectories, gridding techniques (e.g., NuFFT^59^, ^60^) combined with trajectory mapping using field probes^61^, ^62^ are needed to accurately measure gradient trajectories and reconstruct the non‐Cartesian data without errors. With each new rapid acquisition technique, associated MRSI reconstruction techniques^51^, ^63^, ^64^, ^65^, ^66^, ^67^ have been introduced to restore spectral data quality that is lost due to each related undersampled encoding strategy. Regardless of the acquisition strategy, PSF‐mediated lipid signal contamination will always remain a concern until MRSI resolutions approach those seen in conventional imaging. Fortunately, all of the state‐of‐the‐art techniques mentioned above are compatible with the suppression strategies presented in this work. To reduce the acquisition time further, a shortened water suppression sequence such as WET^68^ or tailored water suppression^43^, ^69^ can be included.

### 4.1 Additional considerations

As seen in other MRSI studies, we have reported metabolite values as ratios to total creatine rather than absolute concentrations. This approach is largely necessitated by the short wavelength of the transmit RF pulse at the Larmor frequency when measuring at an ultra‐high field resulting in an inhomogeneous transmit magnetic field (B_1_). The spatial variability in B_1_ can lead to T1‐dependent weighting between different metabolites. In the same vein, differences in the spatial receive profiles of various coil elements may also have an effect on regional metabolite contrast. As described by Klauser et al.,^45^ and also present in our images (Figure 5), these effects can lead to apparent metabolite gradients. To correct this gradient issue, advanced reconstruction methods such as low‐rank TGV, which take into consideration irregular receive profiles, can be implemented.^45^, ^47^, ^70^ Addressing data variability, resulting from factors such as lipid contamination, acquisition‐dependent artifacts and reconstruction methods, is essential to promote wider clinical adoption of MRSI. That being said, the EPSI readout has been used to investigate metabolic aberrations in various brain disorders such as brain tumors,^71^, ^72^, ^73^, ^74^ amyotrophic lateral sclerosis,^75^ schizophrenia,^76^ and dyslexia.^77^ Through further refinement of current technologies combined with innovations in ultra‐high field MRSI, non‐RF–based lipid suppression techniques may become an integral part of MR‐based clinical investigations going forward.

## 5 CONCLUSION

This study demonstrated the feasibility of combining 2D FID‐EPSI acquisition with external lipid crushing and the L2‐regularization algorithm to minimize extra‐cranial lipid signal contamination to deliver good quality MR spectra of the brain. Therefore, our accelerated MRSI technique with extensive in‐plane coverage provides a tool to successfully map neuro‐chemical distributions at 7 T.

## Supporting information


**Figure S1.** Quality assurance maps and the reconstructed metabolite ratio maps divided by tCr of tNAA, tCho, Glx, and mI + Gly generated using different lipid suppression strategies in volunteer 2 (V2) and volunteer 3 (V3). For visualization, T1‐weighted image and all metabolite ratio maps were interpolated by a factor of two (the final matrix size is 76 x 76).
**Figure S2.** Anatomical images with 3 different ROI each volunteer (Volunteer 2 and 3)
**Figure S3.** Visualization of signal pattern from (un)suppressed lipid signal in each ROI (R1, R2, and R3) per volunteer (V1, V2, and V3). Note that each ROI was indicated in Figure 4 (A, bottom) for V1 and Figure S2 for V2 and V3.
**Figure S4.** (A, B) Low‐resolution T1‐weighted images (V2 and V3, respectively) were shown with/without lipid suppression using a crusher coil. (C, D) Lipid contamination maps were generated as a matrix of 38 × 38 voxels. (E, F) Lipid signal leakage near the skull is visible in lipid maps where the intensity scale is reduced by a factor of 10. Note that lipid suppression in V2 and V3 is lower than in V1; however, this is the result of a trade‐off in coverage of cortical regions versus crusher strength.
**Figure S5.** MR spectra obtained from (un)suppressed lipid signal of the two voxels were shown in Figure 4(A,B) with LCModel fit.
**Table S1**. Selected number of voxels each metabolite inside brain
**Table S2**. Lipid area (signal integration) in each ROI per subject

## Data Availability

The data that support the findings of this study are available from the corresponding author upon reasonable request.
