# Supplementary material for: Proton metabolic mapping of the brain at 7 T using a two‐dimensional free induction decay–echo‐planar spectroscopic imaging readout with lipid suppression
Source: NMR Biomed. 2022 May 26;35(10):e4771. doi: 10.1002/nbm.4771 (PMC9541868; doi:10.1002/nbm.4771)

**Supplementary Material**

**
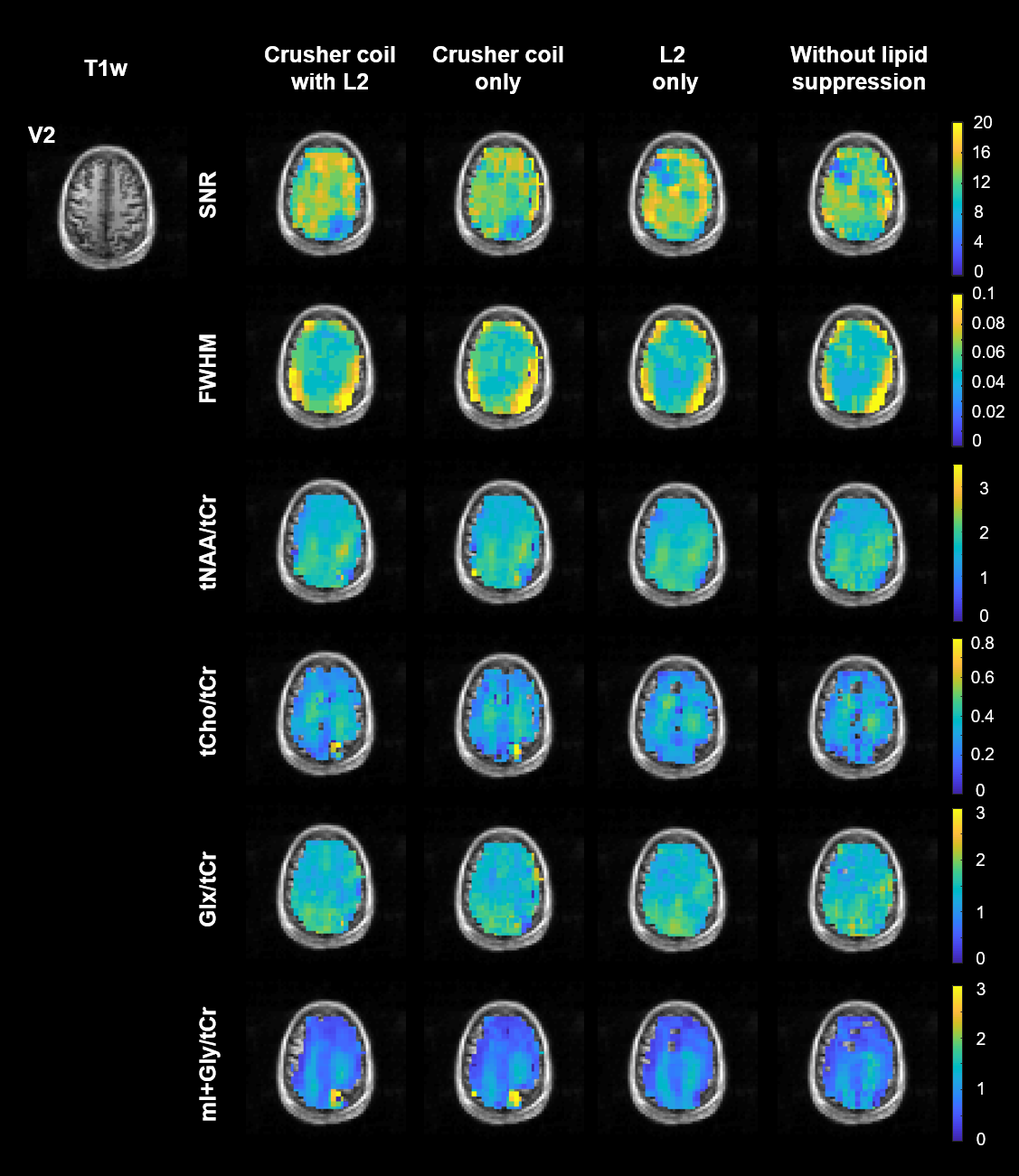
**

**
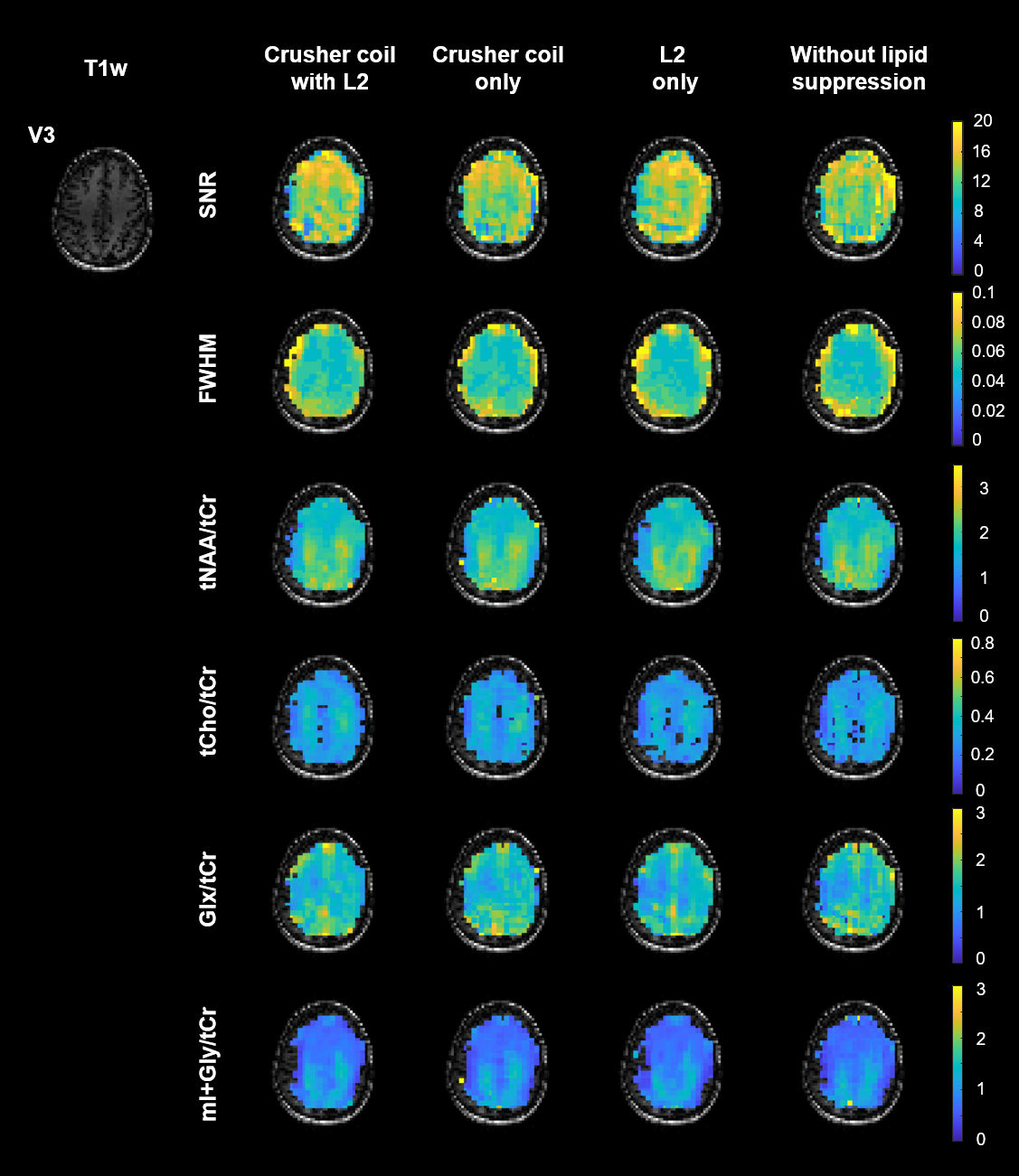
**

**Figure S1.** Quality assurance maps and the reconstructed metabolite ratio maps divided by tCr of tNAA, tCho, Glx, and mI+Gly generated using different lipid suppression strategies in volunteer 2 (V2) and volunteer 3 (V3). For visualization, T1-weighted image and all metabolite ratio maps were interpolated by a factor of two (the final matrix size is 76 x 76).


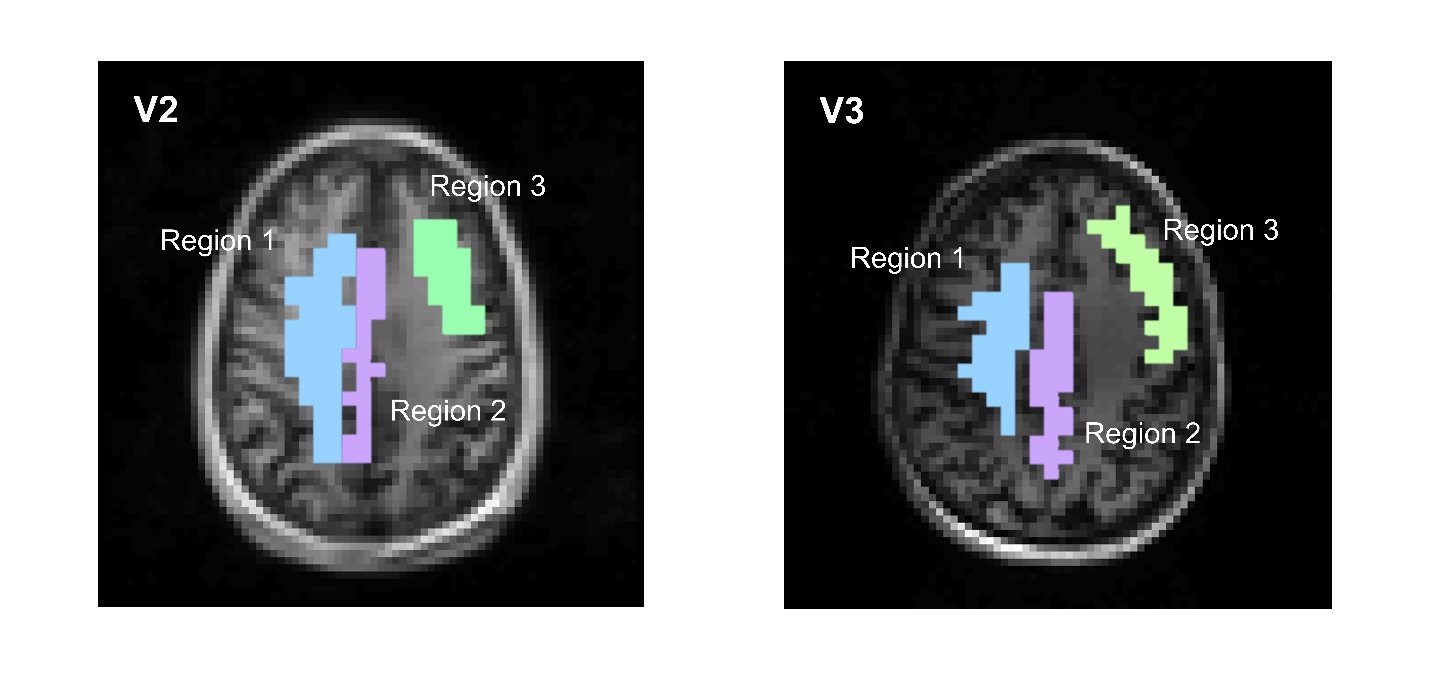


**Figure S2.** Anatomical images with 3 different ROI each volunteer. (Volunteer 2 and 3)


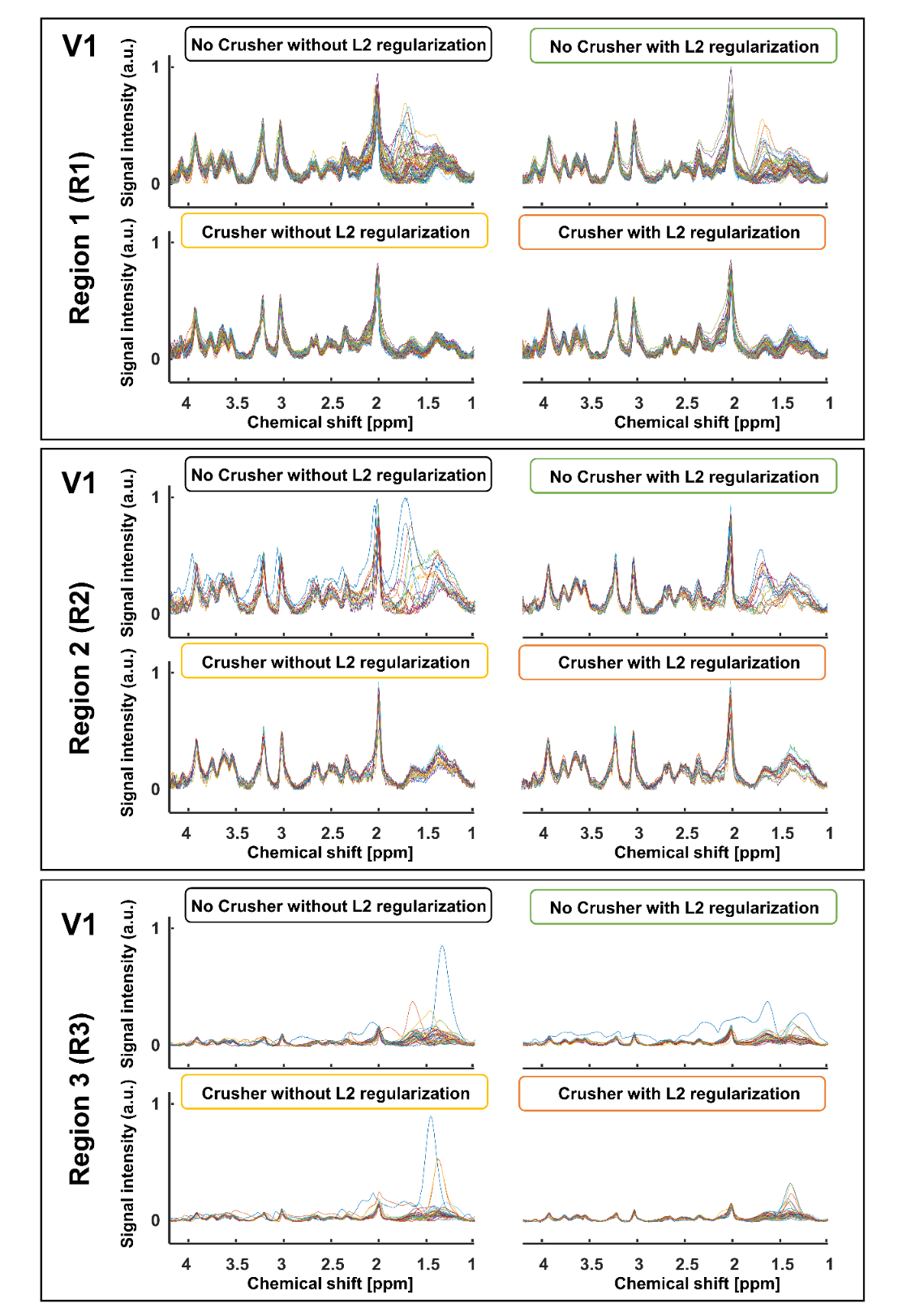


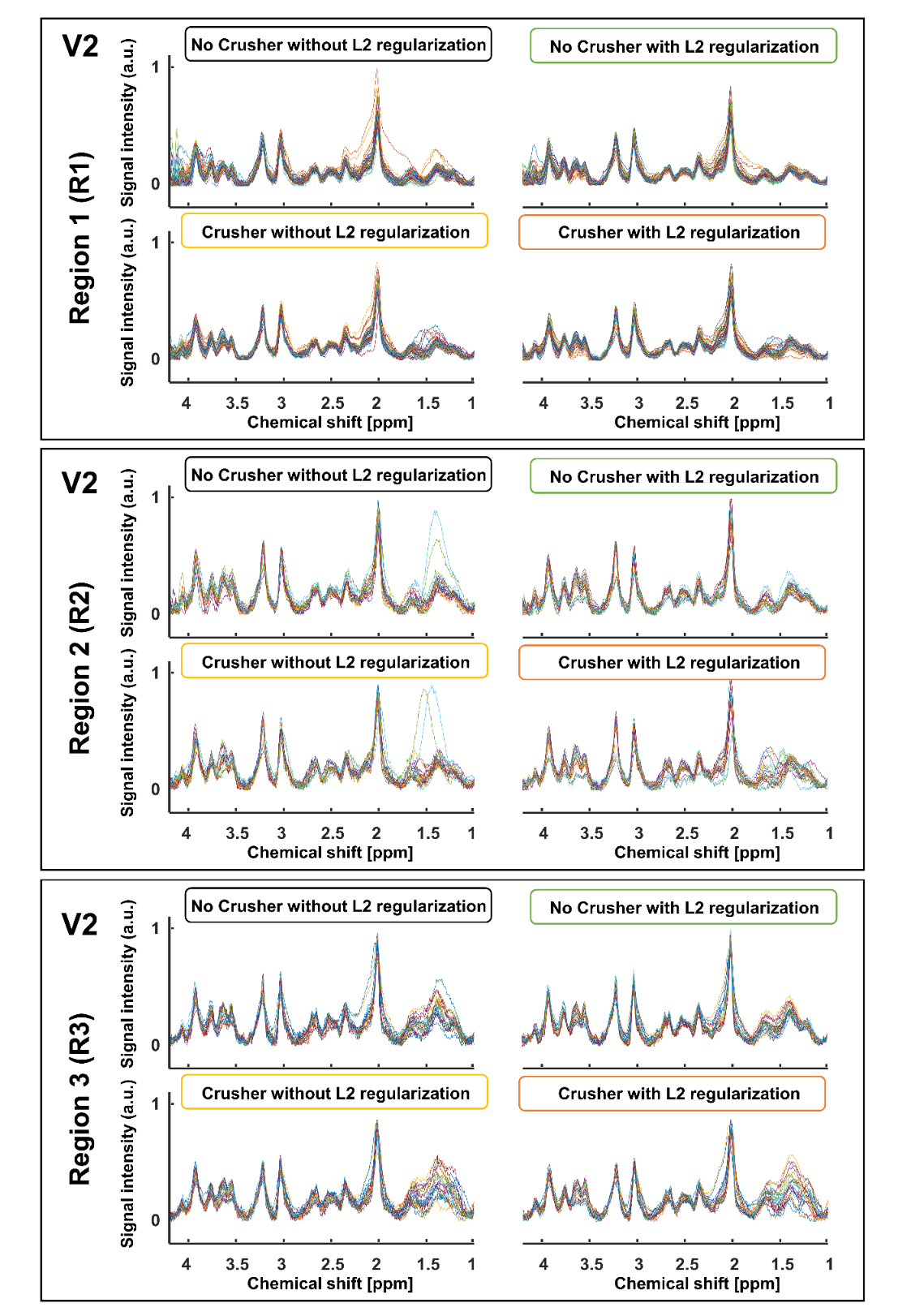


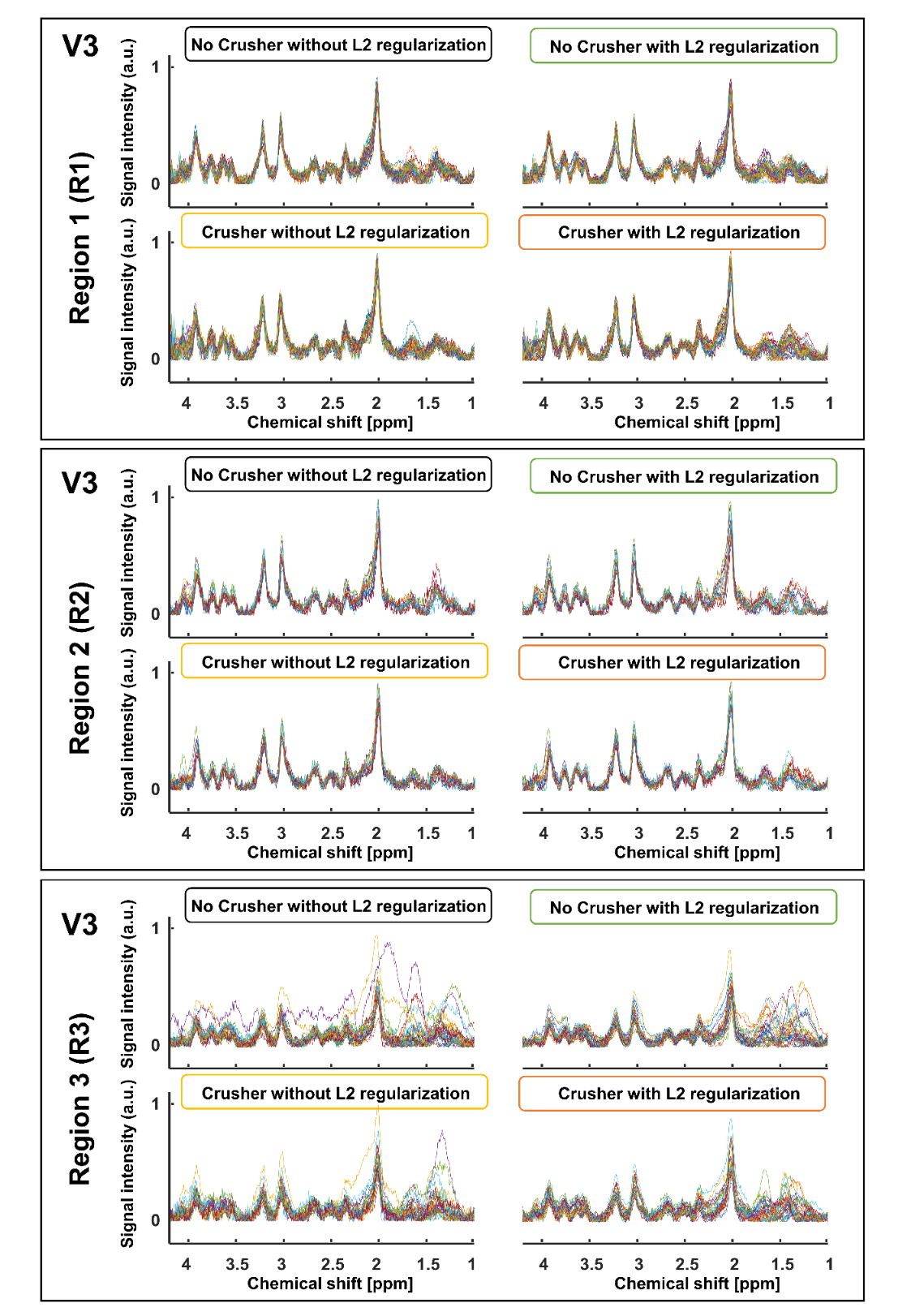


**Figure S3.** Visualization of signal pattern from (un)suppressed lipid signal in each ROI (R1, R2, and R3) per volunteer (V1, V2, and V3). Note that each ROI was indicated in Figure 4 (A, bottom) for V1 and Figure S2 for V2 and V3.


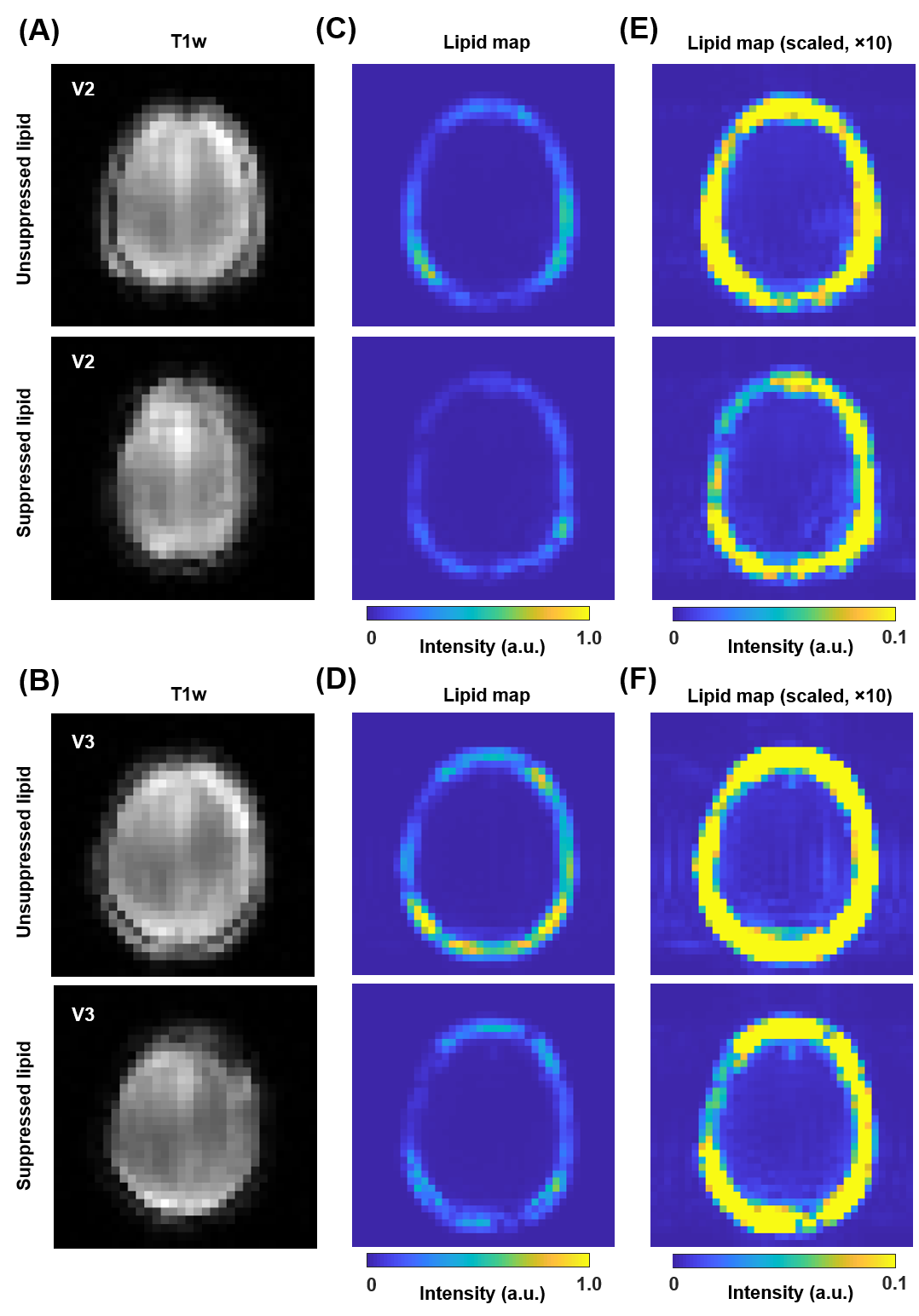


**Figure S4.** (A, B) Low-resolution T1-weighted images (V2 and V3, respectively) with/without lipid suppression using a crusher coil. (C, D) Lipid contamination maps in a matrix of 38×38 voxels. (E, F) Lipid signal leakage near the skull is visible in lipid maps where the intensity scale is reduced by a factor of 10. Note that lipid suppression in V2 and V3 is lower than in V1; however, this is the result of a trade-off in coverage of cortical regions versus crusher strength.


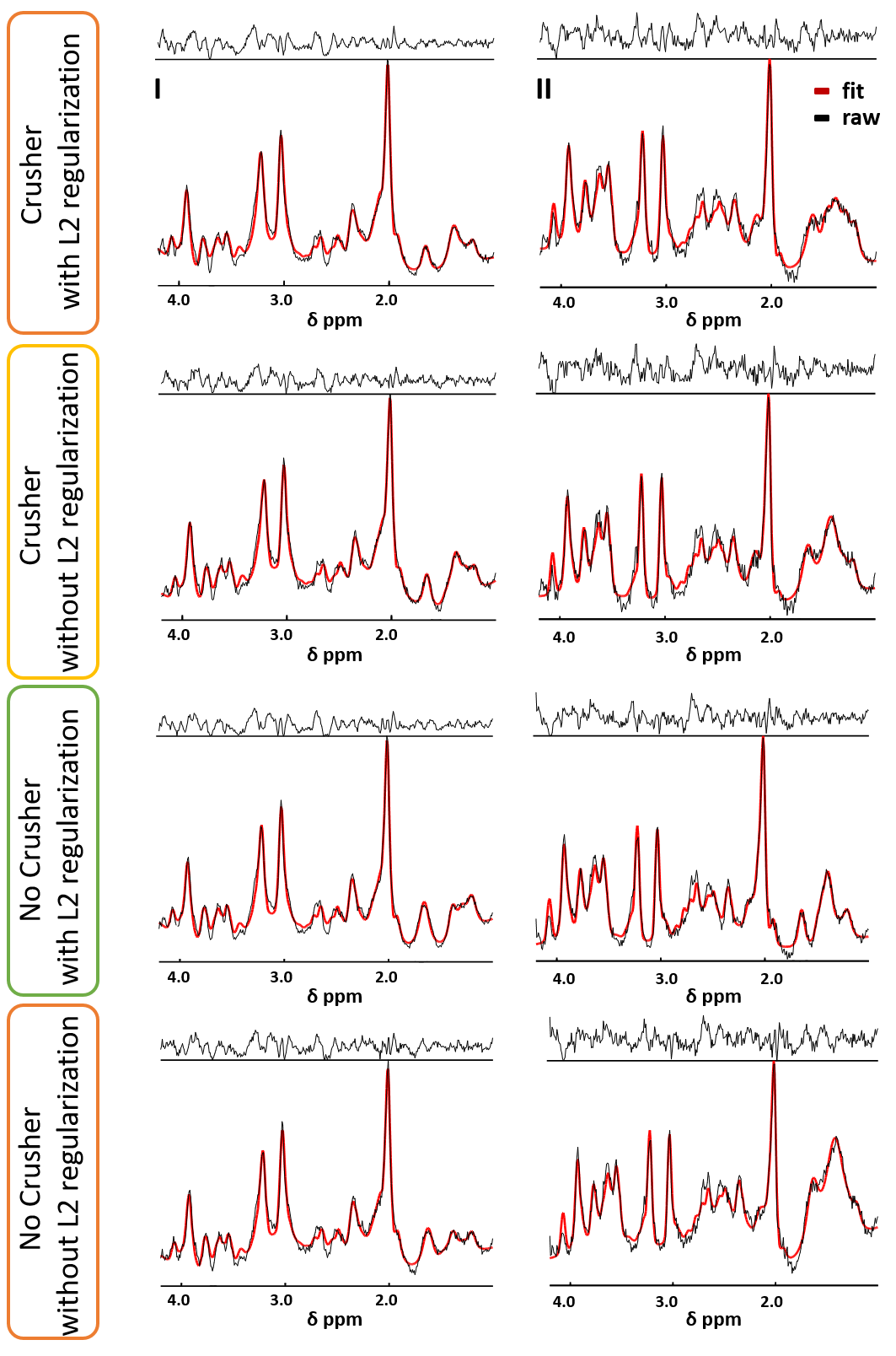


**Figure S5.** MR spectra obtained from (un)suppressed lipid signal of the two voxels shown in Figure 4(A,B) with LCModel fit.


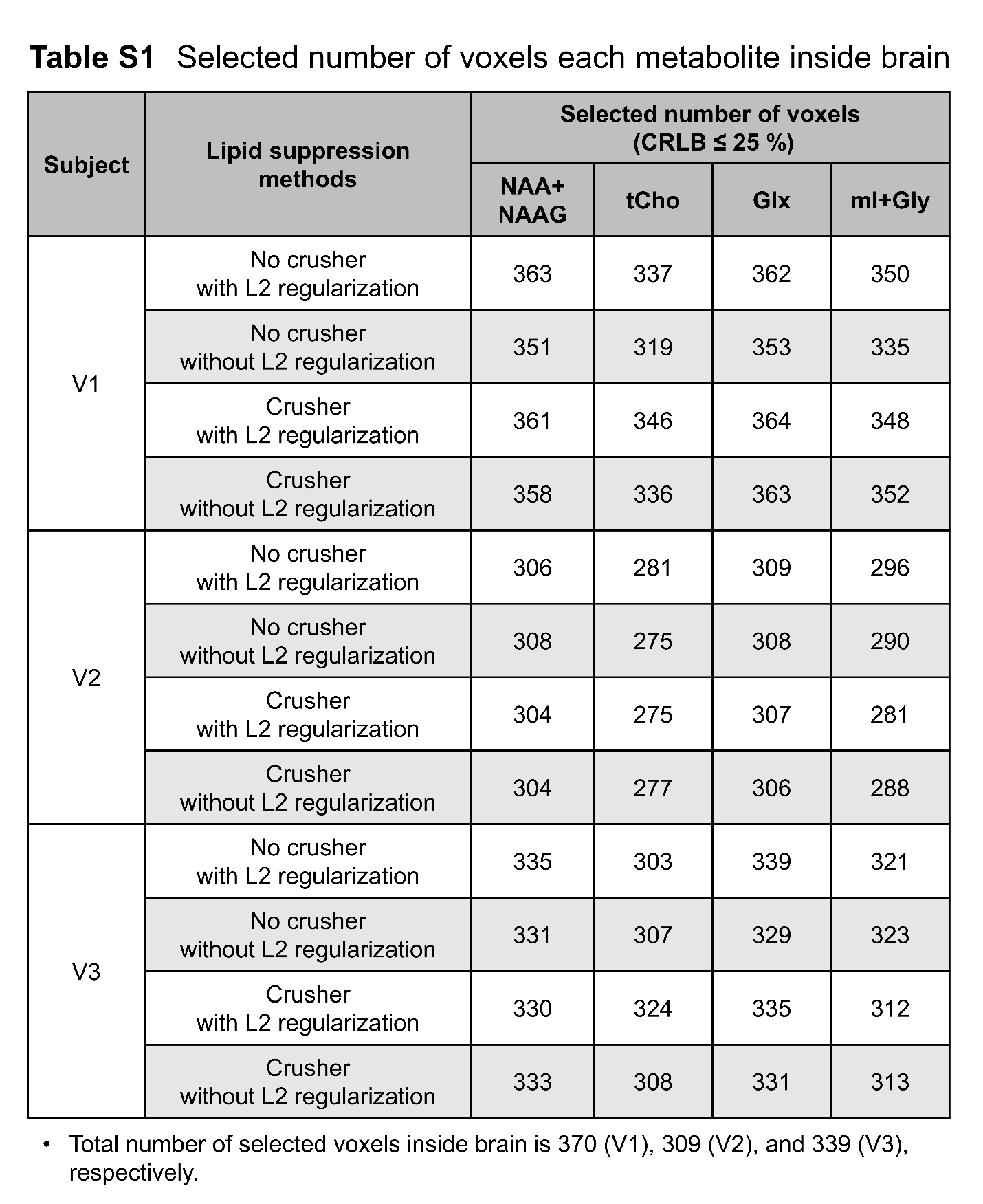


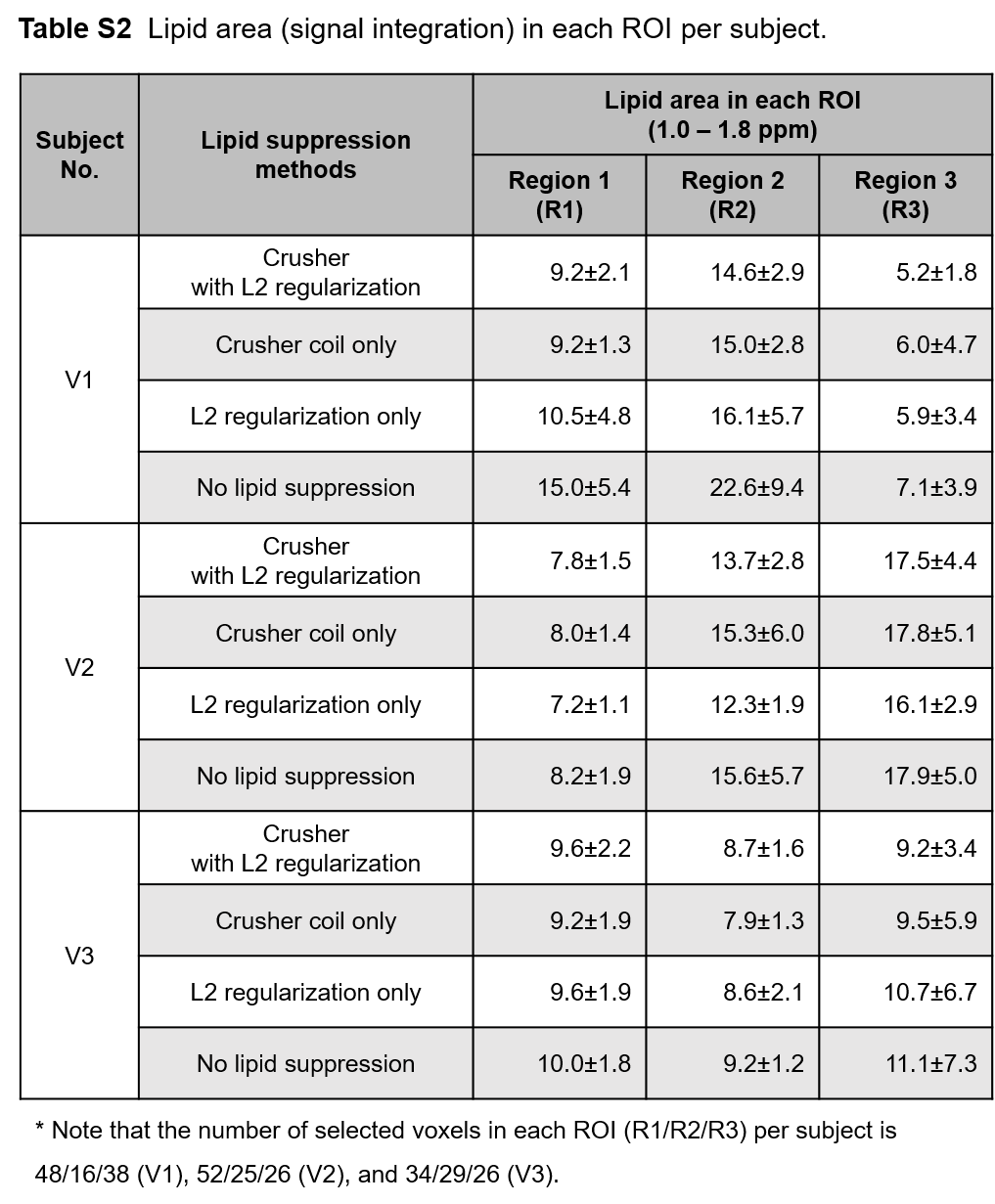

Supplement: Supplementary file 1 — Figure S1. Quality assurance maps and the reconstructed metabolite ratio maps divided by tCr of tNAA, tCho, Glx, and mI + Gly generated using different lipid suppression strategies in volunteer 2 (V2) and volunteer 3 (V3). For visualization, T1‐weighted image and all metabolite ratio maps were interpolated by a factor of two (the final matrix size is 76 x 76). Figure S2. Anatomical images with 3 different ROI each volunteer (Volunteer 2 and 3) Figure S3. Visualization of signal pattern from (un)suppressed lipid signal in each ROI (R1, R2, and R3) per volunteer (V1, V2, and V3). Note that each ROI was indicated in Figure 4 (A, bottom) for V1 and Figure S2 for V2 and V3. Figure S4. (A, B) Low‐resolution T1‐weighted images (V2 and V3, respectively) were shown with/without lipid suppression using a crusher coil. (C, D) Lipid contamination maps were generated as a matrix of 38 × 38 voxels. (E, F) Lipid signal leakage near the skull is visible in lipid maps where the intensity scale is reduced by a factor of 10. Note that lipid suppression in V2 and V3 is lower than in V1; however, this is the result of a trade‐off in coverage of cortical regions versus crusher strength. Figure S5. MR spectra obtained from (un)suppressed lipid signal of the two voxels were shown in Figure 4(A,B) with LCModel fit. Table S1. Selected number of voxels each metabolite inside brain Table S2. Lipid area (signal integration) in each ROI per subject [file NBM-35-e4771-s001.docx]
